# Nanoparticles Loaded with Platinum Drugs for Colorectal Cancer Therapy

**DOI:** 10.3390/ijms231911261

**Published:** 2022-09-24

**Authors:** Buhle Buyana, Tobeka Naki, Sibusiso Alven, Blessing Atim Aderibigbe

**Affiliations:** Department of Chemistry, University of Fort Hare, Alice 5700, Eastern Cape Province, South Africa

**Keywords:** colorectal cancer, nanoparticles, platinum, colon, rectum, anticancer drugs

## Abstract

Colorectal cancer is a common cancer in both men and women. Numerous studies on the therapeutic effectiveness of nanoparticles against colorectal cancer have been reported. Platinum treatments as well as other medications comprising of nanoparticles have been utilized. Drug resistance restricts the use of platinum medicines, despite their considerable efficacy against a variety of cancers. This review reports clinically licensed platinum medicines (cisplatin, carboplatin, and oxaliplatin) combined with various nanoparticles that have been evaluated for their therapeutic efficacy in the treatment of colorectal cancer, including their mechanism of action, resistance, and limitations.

## 1. Introduction

Colorectal cancer (CRC) is one of the most common cancers and is also the second most common cause of cancer death. Over 1.8 million new cases of CRC and 900,000 deaths are reported each year [1,2]. CRC is a cancerous growth that originates from the colon’s epithelial and glandular cells. Patients with ulcerative colitis and Crohn’s disease have a high risk of developing CRC. Other factors that make people prone to developing CRC are smoking due to carcinogenic nicotine, alcohol consumption (i.e., alcohol contains acetaldehyde), a carcinogenic and unhealthy diet, and genetics and epigenetics [3,4]. Complete removal, targeted therapy, neoadjuvant chemotherapy, local ablation, intra-arterial chemotherapy, adjuvant chemotherapy, radiation and immunotherapy all are available for treating CRC [5,6,7]. Colorectal carcinomas are categorized as sporadic (70%), depending on the source of the mutation (25%), and hereditary (5%). The majority of the CRC cases are reported in Western countries and the incidence of the disease is escalating dramatically [8]. CRC is classified into five stages based on the degree of the depth of local invasion, metastasization, and lymph node involvement, with the earliest stage being stage 0 and stage IV being the advanced stage. A poor prognosis, as well as inaccessibility to treatment interventions, is directly tied to the advanced stage. Individuals with stage II cancer and beyond require a fusion of chemotherapy, radiotherapy, and/or surgery to optimize treatment outcomes [3,9]. However, surgical extraction easily removes the tumour at stage 0. As previously stated, the treatment for CRC is determined by the tumour’s characteristics, and the combination of chemotherapy and surgical procedures is frequently chosen. Chemotherapy is still the most common treatment option for CRC. 

The utilization of nanoparticles (NPs) is generally effective for the targeted transportation of active bioactive agents [10]. NPs are very small materials that range between 1 and 100 nm in terms of their diameter. Compared to free medication dosages, this technique allows for more drug intratumoral delivery and less systemic cytotoxicity [11,12]. Micelles, gold NPs, liposomes, polymeric NPs, phytosomes, dendrimers, and magnetic NPs have all been investigated and have been reported to enhance drug–water solubility, enabling disease-specific transportation of drugs [13,14]. Drug administration using the oral route is one of the well-known routes because of numerous advantages, which include: (1) it can be self-administered and is painless, modifying patient compliance; (2) it is extremely the simplest way of administering a drug; (3) it is appropriate for patients who are required to take the drug frequently for a prolonged period; and (4) sterile precautions are not necessarily required [15,16,17,18]. Furthermore, this administration method is suitable for a variety of therapeutic molecules, ranging from small molecules to enormous biomacromolecules. Most importantly, the oral route of drug administration is very desirable for localized medications for several diseases that affect the gastrointestinal system (GIT).

**Figure 1:**
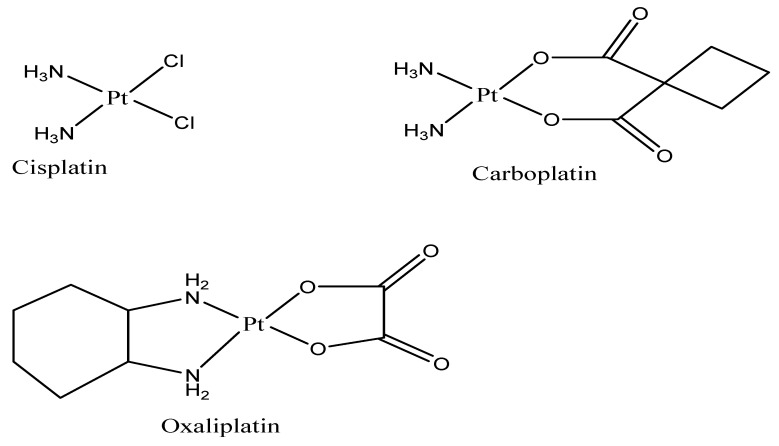
Approved platinum drugs in clinical use.

**Figure 2:**
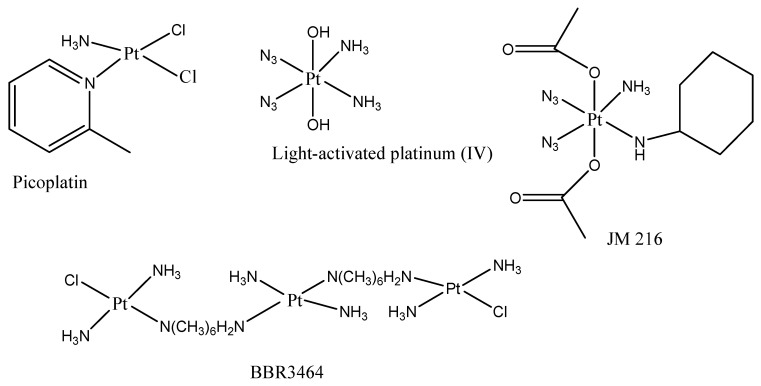
Unclassical platinum drugs that have been formulated.

The class of drugs used for the treatment of CRC are platinum drugs (Figure 1) which are combined with other classes of anticancer drugs. Platinum drugs are also used for the treatment of other types of cancers, including breast, ovarian, lung, etc. Although they are highly effective against a range of cancers, they suffer from drug resistance and significant adverse side effects [19]. The following factors are responsible for platinum drug resistance: (1) decreased cellular uptake of the drug [20], (2) accelerated detoxification [21], (3) enhanced process of Deoxyribonucleic acid (DNA) repair [22], and (4) changed cell signalling pathways and reduced apoptosis [23]. The non-classical platinum therapeutics (Figure 2) such as platinum (IV) drug JM216 that is chemically reductive, picoplatin, multinuclear platinum drug and BBR3464 photosensitive platinum (IV), which target the DNA and inhibit the process of DNA repair more effectively than cisplatin, were developed to overcome drug resistance. Platinum compounds have played a prominent part in cancer treatment and are now extensively utilized to manage head, colorectal, ovarian cancer, neck and non-small-cell lung cancers [24,25]. Platinum drugs have been employed in combination chemotherapy administration with other known drugs, including doxorubicin [26], paclitaxel [27], and gemcitabine [28], as well as radiotherapy [29], accounting for nearly half of all chemotherapy agents used for cancer. The commonly used platinum drugs are cisplatin, oxaliplatin, and carboplatin (Figure 1) which are approved worldwide [30,31,32].

To overcome the drug resistance of platinum drugs, the development of new antitumor agents has been reported by several researchers. Cisplatin or cis-diaminedichloroplatinum (II) derivative was the first to be successfully utilized as an anticancer treatment against a variety of tumours. Cisplatin’s mode of action is via interaction with DNA bases, causing DNA damage and apoptosis. Despite its effectiveness, its unspecific DNA targeting has significant side effects, including neuro- and nephrotoxicity. To preserve the drug’s potent anticancer properties while minimizing its toxicity, a variety of cisplatin analogues have been developed [33].

Carboplatin is a second-generation platinum drug that has a broad scope of antitumor activity. The platinum metals are complexed with a cyclobutane-dicarboxyl backbone and two ammonia groups in carboplatin. The compound is activated intracellularly, generating platinum complexes that are reactive, binding to the nucleophilic groups of the DNA, which include interstrand DNA cross-links, GC-rich sites, and triggering intrastrand as well as DNA-protein cross-links. Cell growth and apoptosis reduction are mediated by protein and carboplatin-induced DNA interactions. This drug has cytotoxic effects that are comparable to its natural substrate, cisplatin, but it is more stable and reduces the adverse effects.

For oxaliplatin, also known as (trans-R,R-cyclohexane-1,2-diamine) oxalatoplatinum(II), a platinum-containing third-generation therapeutic in some cancers, cross-resistance with cisplatin has been reported [34]. It induces intrastrand crosslinks, DNA-protein crosslinks, and also interstrand crosslinks with cellular DNA. It exhibits similar cytotoxic effects to cisplatin but is more effective against some cisplatin-refractory cell lines [35]. Oxaliplatin induces a myelotoxic effect that is moderate when compared to cisplatin with other side effects, such as nausea, diarrhoea, peripheral neuropathy, and vomiting [36,37]. 

## 2. Mechanism of Action of Platinum Drugs

Platinum drugs are recognized as the most effective antitumor therapeutics, although their mechanism of action is not well understood. The platinum drugs that are currently commercially available and clinically employed worldwide are cisplatin, carboplatin, and oxaliplatin [38]. Four platinum-based agents have regulatory endorsement in some countries: lobaplatin (China), heptaplatin (Korea), nedaplatin (Japan), and miriplatin (Japan) [39]. The molecular structures of some platinum drugs are shown in Figure 1. Cisplatin (cis-diamminedichloroplatinum (II)) is a well-known anticancer drug that has been utilized for the treatment of various human cancers including testicular, ovarian, neck and head, lung, and bladder cancers [40]. This anticancer drug is also effective against many types of cancers, including germ cell tumours, carcinomas, sarcomas, and lymphomas. The mechanism of action of cisplatin has been associated with its ability to crosslink with purine bases on the deoxyribonucleic acid (DNA), interfering with DNA repair mechanisms, leading to DNA disruption, and stimulating apoptosis in cancer cells [41]. 

Carboplatin (1,1-cyclobutyldicarboxylate) is a cisplatin derivative with a similar mode of action to cisplatin but differs in relation to molecular structure and toxicity [42]. Carboplatin was approved by the Food and Drug Administration (FDA) during the 1980s and since then, it was broadly employed for the treatment of various types of cancer (ovary, testis, neck, head, and small-cell lung cancer) [43]. This anticancer agent produces lesions in DNA via the development of adducts with platinum, thereby resulting in the inhibition of replication and transcription, causing cancer cell death. The form of these adducts affects most of the transduction pathways and induces necrosis or apoptosis in cancer cells. The adducts produced by carboplatin can be either in the form of inter- and intrachain diadducts or monoadducts [43].

Oxaliplatin (trans-l-diaminocyclohexane oxalate platinum (II)) is the third-generation platinum drug that was developed to offer an improved safety profile in comparison with first- and second-generation platinum drugs [44]. This anticancer drug was approved by the FDA in 2002 to treat CRC although it was introduced in the 1970s. Oxaliplatin is regularly used for the treatment of breast, ovarian, colorectal, pancreatic, indolent lymphoma, acute myeloid leukaemia, fallopian tube, hepatoma, oesophagogastric, and non-small-cell lung cancer (NSCLC) [45]. The order and region specificity of oxaliplatin-DNA adducts are similar to cisplatin. Oxaliplatin exhibits less reactivity than cisplatin, but it displays efficacy to inhibit DNA biosynthesis. Thus, oxaliplatin often displays an influence on cisplatin-resistant tumours [46]. 

## 3. Mechanism of Resistance and Limitations of Platinum Drugs 

Although platinum drugs demonstrate good anticancer activity against numerous types of cancer, they also suffer from drug resistance and toxicity like other anticancer drugs. The rapid degradation of the copper membrane transporter CTR1 by cisplatin in human cells reduces the influx of cisplatin, resulting in drug resistance [47]. Some cancer cell lines such as colon, hepatocellular, ovarian, and bladder that develop resistance to cisplatin exhibit an overexpressed multidrug-resistance-associated protein 2 (MRP 2) level [48]. The concentration of cisplatin in the proximal tubular epithelial cells has been reported to be five times more than in the serum. This excess cisplatin that accumulates in the kidney tissue induces nephrotoxicity. Nephrotoxicity is the most common dose-limiting toxicity associated with cisplatin treatment that limits its clinical application [49]. The following features associated with cisplatin-induced kidney toxicity are cellular atypia with the development of multinucleated giant cells, interstitial inflammatory cell infiltration, tubular dilatation, interstitial fibrosis, epithelial cell necrosis, and thickening of the tubular basement membrane [50].

The mechanism of resistance of carboplatin is very similar to that one of cisplatin. The copper transporters, copper-transporting P-type ATPase/Menkes ATPase (ATP7A), copper-transporting ATPase 2 (ATP7B), and CTR1 are responsible for the drug resistance of carboplatin. CTR1 controls the accumulation of anticancer drugs in cells based on platinum [50]. The study conducted by Holzer and co-workers evaluating the influence of CRT1 on carboplatin accumulation showed that the drug concentration was increased significantly, exhibiting its dependency on CTR1 [51]. Platinum-based anticancer agents also induce haematological toxicity that influences blood cell production and bone marrow function, bleeding conditions, and anaemia and predisposes the patient to chronic infections. Although carboplatin can cause haematological toxicity, its effect is reduced when compared to cisplatin, making carboplatin a suitable drug for higher-dose chemotherapy in some types of cancers [50].

The knockout of CTR1 from the cells causes an important effect on cells at a low concentration of oxaliplatin, whereby there are almost no side effects in the cells visible at high concentrations of oxaliplatin demonstrating that the uptake of oxaliplatin is not depending on CRT1 at high concentrations [51]. Additionally, P-glycoprotein (P-gp) plays a crucial role in reducing the intracellular accumulation of anticancer complexes and the overexpression of P-gp was observed in oxaliplatin-resistant colorectal tumour cells [52]. Oxaliplatin induces peripheral sensory neurotoxicity, limiting its application in the treatment of cancers. Oxaliplatin-induced peripheral sensory neurotoxicity can be chronic or acute [53]. The signs of acute neurotoxicity include pharyngolaryngeal dysesthesias, cold-related transient paresthesia, cramps, jaw spasms, and muscular spasms in the limbs. Chronic neurotoxicity happens in a dose-dependent manner resulting from oxaliplatin-induced DNA adduct accumulation in the dorsal root ganglia [53].

### Clinical Trials of Platinum-Based Drugs in CRC

In the past 30 years, hundreds of platinum analogues have been developed, and the interest in this approach has grown. However, there have been reports of clinical failures, such as tetraplatin. Clinical trials on oxaliplatin in patients with colorectal cancer showed limited single-agent efficacy (10% objective response rate from >100 patients) [54], but more encouraging activity was significant when combined with 5-fluorouracil (5FU) and leucovorin (LV) (58% objective response rate from 93 patients) [55]. Patients in the first of these studies received 5FU with LV with or without oxaliplatin as first-line therapy [56] for advanced colon cancer. Oxaliplatin was introduced, and this significantly increased antitumor effectiveness (median progression-free survival of 8.7 months compared to 6.1 months without; *p* = 0.048). Another study employed bolus 5FU with LV followed by a 22 h infusion of 5FU alone or with oxaliplatin [57] in patients with previously untreated colon cancer. However, the group receiving oxaliplatin had a superior response rate (50.7% versus 22.3%, *p* = 0.0001) and a significantly longer median progression-free survival (9.0 versus 6.2 months, *p* = 0.0003). In a third trial, patients were given bolus and infusion of 5FU and LV (LV5FU2), single-agent oxaliplatin, or the combination of LV5FU2 and oxaliplatin if they had progressed on bolus 5FU with LV and irinotecan (Camptosar, Pfizer; IFL) (FOLFOX4). According to the objective response rate (9.9% versus 0% for LV5FU2, *p* = 0.0001) and median time to progression (4.6 months versus 2.7 months for LV5FU2, *p* = 0.0001) [58], the FOLFOX4 regimen demonstrated considerably greater clinical activity. Patients with metastatic colorectal cancer who had not previously received treatment either received IFL or oxaliplatin and 5FU with LV (FOLFOX) or irinotecan and oxaliplatin (IROX). The FOLFOX arm showed significantly greater response rates, longer median durations to progression, and longer median survival periods (for instance, a median survival of 19.5 months against 15.0 months for IFL and 17.4 months for IROX) [59].

To treat advanced MC38 murine colon cancers, Fu et al. [60] reported using a sequential therapy of PD-1 blockade therapy (anti-PD-1 antibody or anti-PD-L1 antibody) in combination with various doses of platinum drugs (cisplatin or oxaliplatin). Despite having no discernible impact on tumour growth, 10 mg/kg of platinum (cisplatin or oxaliplatin) in combination with the sequential injection of anti-PD-1 antibodies resulted in a full tumour remission in 80–100% of mice. According to the research, low-dose (10 mg/kg) platinum treatment enhanced the T cell response by increasing the number of peripheral T cells, while high-dose (20 mg/kg) platinum treatment caused lymphopenia in mice with the MC38 tumour. Notably, three days after 10 mg/kg oxaliplatin treatment, greater numbers of PD-1 positive CD8 T cells were discovered in draining lymph nodes, peripheral blood, and tumour tissues. Additionally, along the edge of tumour tissues, more CD8 T cells and apoptotic tumour cells were found. Further research revealed that platinum-compound-induced tumour cell death boosted T cell activation. Moreover, after platinum treatment, MC38 cells showed enhanced production of the chemokines that draw T lymphocytes (CXCL9, CXCL10, and CCL5). These findings suggested that successive PD-1 inhibition could prevent freshly arriving T cells from being tired in tumour sites and that the optimum dose of platinum chemotherapy could activate and recruit T cells into tumours. These results show the value of tailoring the timing and dose of platinum chemotherapy in combination with PD-1 blocking and give a hint for the development of combination treatments in clinical trials [60]. 

Petrelli et al. investigated the anticancer activity of the combination of 5-fluorouracil (5-FU) and cisplatin in metastatic colorectal carcinoma. To achieve comparable activity without the nephrotoxicity seen with 5-FU/cisplatin, the combination of 5-FU and iproplatin was explored. Although, with 5-FU/iproplatin, no reactions were obtained [61].

In comparing CHIP and carboplatin for the treatment of previously untreated metastatic colorectal cancer, Asbury et al. [62] reported clinical studies of 56 patients who were given treatment in each of the study’s two arms. With CHIP, there was just one partial reaction (2%) while with carboplatin, there were two partial responses (4%), and when compared to carboplatin, the side effects of CHIP were much more severe. Vomiting and hematologic side effects were the two conditions for which both medications were most frequently used. Life-threatening side effects were experienced by 16% of patients administered CHIP and 9% of patients administered carboplatin. Both medications are ineffective against metastatic colorectal cancer [62]. The effectiveness of carboplatin (CBDCA) was investigated by Nole et al. in 21 consecutive patients with advanced colorectal cancer that had advanced while receiving fluoropyrimidine therapy. Given the positive outcomes of earlier phase II investigations, CBDCA was selected. The 400 mg/m^2^ dose of the chemical, which was administered every 21 days, failed to show any signs of action. Haematological toxicity was the main problem. CBDCA was not advised for colorectal cancer patients who had already had treatment [63]. In a study by Britzer et al., 19 patients with colorectal cancer who had not previously received treatment and had a quantifiable illness were given 75 mg/m^2^ of iproplatin (CHIP) daily for 5 days every 4 weeks for at least two courses [64]. Myelosuppression, moderate nauseousness, and uncommon mild nephrotoxicity were among the toxicities. Thrombocytopenia, which seemed to be cumulative, was the hazardous effect that was dose-limited. It was frequently essential to reduce the dose. No toxic deaths occurred. Four patients had stable illness after a median of two months while one partial response was seen. Iproplatin does not seem to have much of an impact on colorectal cancer [64]. Han et al. researched liposomal cis-bis-neodecanato-trans-R,R-1, diaminecyclohexaneplatinum (11) (L-NDDP), a platinum complex that is entrapped in a liposome and has partially demonstrated a lack of cross-resistance with the chemotherapy drug cisplatin in human colon cancer LoVo cells. In LoVo and LoVo/PDD cells, we investigated drug accumulation and DNA damage brought on by L-NDDP and cisplatin [65]. Our findings show that cisplatin accumulates more slowly than L-NDDP in LoVo cells, that L-NDDP accumulates similarly in both cell lines while cisplatin accumulates more slowly in LoVo/PDD cells, and that cisplatin’s transmembrane transport is strongly influenced by temperature while L-is NDDP’s not. They also discovered that DNA interstrand cross-linking does not appear to play a function in the cytotoxicity of L-NDDP, although it does play a role in the cytotoxicity of cisplatin, and that the degree of DNA-protein cross-link formation correlates with the cytotoxicity of both drugs [65].

## 4. Nanoparticles Loaded with Platinum Drugs for Colorectal Cancer Treatment 

### 4.1. Nanoparticles Loaded with Cisplatin

The fundamental formula for platinum compounds is cis-[PtX_2_ (Am)_2_], where X is the leaving group and Am is an inert amine with one stable N-H moiety. Polymeric (AP5280 [66], AP5346 [67,68,69], and NC-6004 [70,71,72,73]), solid lipids (lipoplatin) [74], and inorganic nanoparticles (AP5280) [75] have all been developed as platinum drug delivery platforms. A broad variety of platinum compounds, comprising platinum (II) [76], platinum (IV) prodrugs [77], photosensitive platinum (IV) drugs [78], and multinuclear platinum drugs [76], have been loaded into different nanocarriers, featuring drug release triggered by pH, redox, or light. Additionally, for enhanced clinical efficacy, selected ligands (folic acid, antibody, and peptide) were incorporated to achieve selective targeting of the malignant cells [78,79,80].

Wheate et al. formulated gold nanoparticles for transporting cisplatin with enhanced effects. Cisplatin was tethered to gold-coated iron oxide nanoparticles for delivery to the tumours [81]. Aquated cisplatin was incorporated into the nanoparticles via a thiolated polyethylene glycol linker. The nanoparticles were in the range of 60–120 nm and were more than 110-fold more cytotoxic on A2780 and A2780/cp70 cancer cell lines than cisplatin [82]. Guo et al. tethered super-paramagnetic iron oxide NPs to carboxylate groups on the surface of the dechlorinated cisplatin [83]. The nanocrystals were highly internalized in the tumour when compared to cisplatin, a unique feature that can overcome drug resistance associated with platinum drugs. The nanocrystal cytotoxicity was compared to cisplatin and was found to be comparable to or higher than cisplatin.

Tao et al. used two types of mesoporous silica microparticles (MSN) (SBA-15 and MCM- 41) with different pore dimensions, particle sizes, and internal diameters to load transplatin and cisplatin separately [84]. The drug-loaded microparticles were less cytotoxic to leukaemia cells than the free drugs, 12 h after exposure and significant after 24 h of exposure. The cytotoxicity of the drug-loaded microparticles indicates a localized intracellular release of the platinum compounds [85]. The transplatin-loaded MSN NPs significantly outperformed the cisplatin-loaded predecessors. This might be because platinum compound nanoparticulate delivery fluctuates the pathway of intracellular uptake and avoids unwanted transplatin detoxification, suggesting the possibility of just using traditionally ineffective or non-toxic chemical compounds for the nanomedicine therapy of cancer. Rieter et al. [86] developed Tb_2_(DSCP)_3_(H_2_O)_12_ NPs where DSCP is disuccinatocisplatin. The nanoparticles displayed extended half-lives of the loaded drug, disuccinatocisplatin, for 9 h. In vitro cancer cell cytotoxicity studies on a human colon carcinoma cell line (HT-29) further showed that internalization of the drug from the particles with enhanced anticancer efficacy, which was superior to cisplatin. Lin et al. utilized simple coupling chemistry to covalently link cisplatin (IV) precursor, ethoxysuccinato-cisplatin to Fe(III)-carboxylate nanoscale metal–organic frameworks [87]. The rate of drug release was influenced by the coating of the particles with a silica shell. The NPs were effective in vitro on HT-29 human colon adenocarcinoma cells with good optical imaging capability. The drug release t1/2 of Pt was enhanced from 1.2 to 14 h when the NPs are further coated with silica. Self-assembly of zinc bisphosphonate nanoscale coordination polymer was loaded with 48 wt% cisplatin prodrug and 45 wt% oxaliplatin prodrug. The NPs exhibited excellent blood circulation half-lives of 12.0 ± 3.9 h and 16.4 ± 2.9, for the NPs loaded with oxaliplatin and cisplatin, respectively, in vivo. In further studies on H460 lung cancer, AsPC-1, CT26 colon cancer, and pancreatic cancer, superior potency was reported compared with the three free drugs [87]. Nanotherapeutics based on nanoscale coordination polymer offers significant advantages, including the use of mild conditions for their synthesis; consistent batch-to-batch particle size and drug loading; high drug loadings overcoming potential aggregation; possessing a near-neutral surface charge, a desirable feature for nanotherapeutics that prevent self-aggregation and phagocytosis^,^ and minimizes non-specific interactions with proteins; small particles size for enhanced uptake into the tumours; extends the circulation times, a key feature that promotes passive targeting by the EPR effect; non-toxicity; biocompatibility; no burst release preventing premature drug release; and the presence of a built-in trigger release mechanism, further improving drug uptake into the tumours [88]. Based on the outstanding features of nanoscale coordination polymer-based nanotherapeutics, they are drug delivery systems for potential translation to clinic use.

### 4.2. Nanoparticles Loaded with Carboplatin

Some research studies reported NPs loaded with carboplatin for the treatment of CRC. Zhu and co-workers formulated amino-functionalized polyphosphazene NPs encapsulated with carboplatin for the treatment of colon cancer [89]. The Fourier-transform infrared (FTIR) and proton nuclear magnetic resonance (^1^HNMR) spectroscopy confirmed the successful amino functionalization of polymeric nanoparticles. The dynamic light-scattering (DLS) analysis of nanoparticles revealed particle sizes that ranged between 150 and 200 nm, which is suitable for drug delivery in the treatment of cancer. The in vitro cytotoxicity studies using an MTT assay demonstrated more antiproliferative activity against colon cancer cell lines (CT-26) with high apoptosis when incubated with carboplatin-loaded NPs than when incubated with drug-free NPs, suggesting good anticancer efficacy of carboplatin-loaded nanoparticles. Furthermore, the in vivo anticancer experiments using murine colon adenocarcinoma CT-26 tumour-bearing BALB/c mice showed that carboplatin-loaded NPs induced a good decrease in tumour growth, with a tumour growth inhibition of about 55.6% [89].

Profirio et al. prepared poly (D, L-lactic-co-glycolic acid)-based NPs loaded with carboplatin using the nanoprecipitation method [90]. The particle size of the nanoparticles was 121.0 nm with a zeta potential of −34.0 mV and polydispersity index (PDI) of 0.120 and the NPs were stable for 2 months. The drug loading was 0.37% with an entrapment efficiency of 5% and NP yield of 77%. The particle size of the folic acid functionalized poly(lactic-co-glycolic acid) (PLGA) NPs was 178.0 nm, zeta potential of 46.0 mV, PDI of 0.20, entrapment efficiency of 35.5%, drug loading of 1.8% and nanoparticle yield of 92%, indicating that folic acid functionalization significantly improved DLS results of PLGA NPs which can lead to good cellular uptake by colon cancer cells [91]. Li et al. formulated phosphonated calixarene NPs co-encapsulated with carboplatin and paclitaxel for colon cancer treatment [91]. The particle size analysis showed that the loading of the drugs increased the particle size of the NPs from 84 ± 8 nm to 119 ± 13 nm with a surface charge of −40.8 ± 8.8 and −35.4 ± 4.2 mV, respectively. The anticancer studies demonstrated that the dual drug-loaded NPs possessed more cytotoxicity against colon cancer cells (HT-29 cells) than colon cancer cells (Caco-2 cells) with IC_50_ of 0.4 ± 0.02 and 2.1 ± 0.3 μM, respectively, with high apoptosis in HT-29 cells (56.6 ± 4.5%) and Caco-2 cells (44.9 ± 3.44%). These results showed the potential of phosphonated calixarene NPs in the delivery of two anticancer drugs to the colon cancer with a more synergistic effect than a single drug [91].

Abdelwahab et al. designed folate-decorated albumin nanoparticles loaded with carboplatin using the desolvation method for colon cancer therapy [92]. The particle size was 267.29 nm, with a negative surface charge of −30.4 mV and a PDI of 0.069. The in vitro cytotoxicity experiments using an MTT assay showed that the folate decorated nanoparticles loaded with carboplatin possessed the superior inhibitory activity on Caco-2 colon cancer cells with an IC_50_ of 89.1 μg/mL, while the IC_50_ of the carboplatin alone was 192.8 μg/mL, indicating the promising anticancer efficacy of folate-decorated nanoparticles loaded with carboplatin against colon cancer [92]. Pairoj et al. formulated zinc oxide (ZnO) NPs co-incorporated with carboplatin and doxorubicin for the treatment of various cancer types (colon, breast, liver, uteri, cervix, and oral cancer). These NPs displayed a loading capacity and efficiency of 77.81% and 99.05%, respectively. The anticancer studies of ZnO NPs co-incorporated with carboplatin and doxorubicin showed good antitumor efficacy against HT-29 cancer cell lines under the influence of UV with an IC_50_ of 0.173 µg/mol and they were selective in their uptake into the target cells where the loaded drugs were released to induce acute apoptosis with a significant absence of a growth signal of target cancer cells [93].

### 4.3. Nanoparticles Loaded with Oxaliplatin

#### 4.3.1. Nanoparticles

NPs are in the range of 1 to 100 nm and are classified based on their features, forms, sizes, and physical and chemical characteristics [94]. They are classified as ceramic NPs, semiconductor NPs, metal NPs, carbon NPs, lipid-based NPs, and polymeric NPs [95].

#### 4.3.2. Lipid-Based Nanoparticles Loaded with Oxaliplatin

LNPs are multicomponent systems containing poly(ethylene glycol) (PEG)-lipid, cholesterol, phospholipid, and amino lipid [96,97]. There are different types of LNPs, such as nanostructured lipid carriers (NLCs), nanoemulsions, SLNs, and liposomes [98]. LNPs have drawn a lot of interest because of their potential to improve therapeutic bioavailability while reducing the adverse effects and drug metabolism susceptibility [99]. The most extensively used nanomedicine platform in cancer therapy, LNPs, is a potential delivery platform for anticancer drugs [100].

#### 4.3.3. Solid Lipid Nanoparticles (SLNs) Loaded with Oxaliplatin

**Figure 3:**
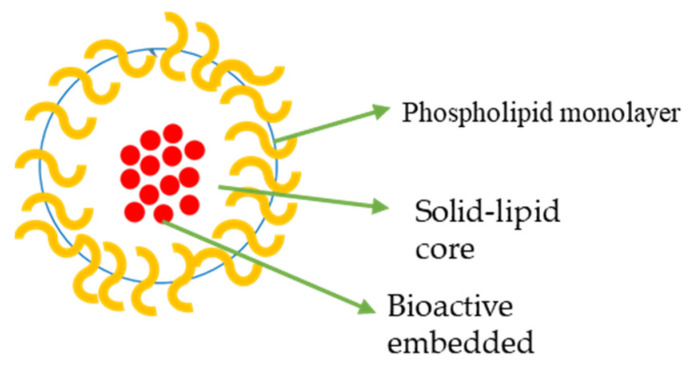
Solid Lipid Nanoparticle.

SLNs are matrix systems in which the drug is physically and evenly disseminated (Figure 3) [101]. SLNPs are mainly composed of low-melting-point lipids and various surfactants and/or co-surfactants [102]. They are utilized to enhance the delivery of drugs that are poorly soluble in water. For instance, quercetin, an antioxidant found in onions, has powerful antitumour properties against CRC, although it is poorly soluble in water [103].

Rajpoot et al. formulated SLNPs comprising tristearin, lipoid S75, and Tween 80, 1,2-distearoyl-sn-glycero-3-phosphoethanolamine [104]. The SLNPs were loaded with oxaliplatin and conjugated with folic acid. The drug-uncoupled SLNPs and drug-loaded folic acid conjugate SLNPs exhibited an entrapment efficiency of 49.2% and 43.5%, respectively. The particle sizes of the NPs were in the range of 146.2 ± 4.4–158.8 ± 5.6 nm with a uniform distribution within the NPs. The drug release profile from the NPs was sustained for 6 days. The anticancer activity of the formulation on HT-29 cell line showed high potency for the drug-loaded folic acid conjugates SLNPs [105]. SLNPs offer several advantages making them appropriate to treat cancer, such as high bioavailability, cost-effectiveness, high biocompatibility, drug targeting, controlled release of the loaded drug, physical stability, etc. Folic acid is an important specific targeting ligand for enhanced intracellular uptake for receptor-mediated endocytosis. It is a known target molecule in cancer drug delivery because it is required for rapid cell growth in many cancer cells [104,106]. Tummala et al. used the micro emulsion method to prepare SLNPs. The SLNPs were effective for the delivery of oxaliplatin to colorectal tumours. The particle size was 127.8 ± 4.4 nm and the release profile was sustained with an initial burst release [107]. Nobili et al. reported a combination of oxaliplatin, 5-fluorouracil, and folinic acid to treat CRC. It is used as adjuvant therapy in the treatment of colon cancer (stage III) [108].

#### 4.3.4. Liposome Loaded with Oxaliplatin

**Figure 4:**
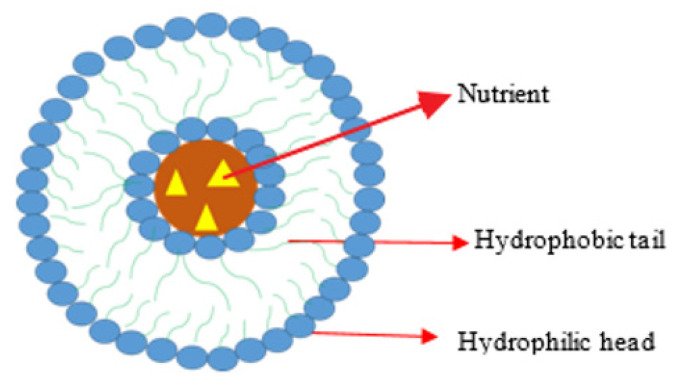
Liposome NP.

Liposomes have been designed by several researchers for drug delivery (Figure 4). Dragovich et al. evaluated the antitumour efficacy of liposomal DACH platinum (L-NDDP) in patients with advanced CRC that were on irinotecan and leucovorin/5-fluorouracil therapies. The toxicity profile and optimal dosing for L-NDDP monotherapy were also evaluated [109]. Twenty patients were enrolled in the study (7 females and 13 males). The outcomes were moderate, with 5.6 percent achieving a partial response, 16.7% reaching a stable disease, and 77.8% of the patients having disease progression due to non-compliance with the study guidelines or intolerance to the dug. The formulation was well tolerated; however, there is a need for further clinical studies on the combination of the formulation with fluoropyrimidines [109]. Ying et al. reported the oxaliplatin which was encapsulated into PEG-coated cationic liposomes [110] and long-circulating liposomes (PEG-liposomal oxaliplatin). The effects of PEG-liposomal oxaliplatin on a tumour formed by a human CRC cell line (SW480) in female BALB/c nude mice were studied. When compared to the free drug in their hydrophobic bilayer, PEG-liposomal-oxaliplatin induced an apoptotic reaction. The uptake of the formulation into the tumour cells due to the liposome degradation increased the intracellular drug delivery and concentration within the cells, thereby inhibiting the drug efflux mechanism. The modification of liposomes with PEG enhances their affinity to the cancer cells and cellular drug uptake [111]. Garrido et al. loaded a combination of oxaliplatin and cetuximab (a monoclonal antibody) in liposomes for the treatment of metastatic CRC expressing epidermal growth factor receptor [112]. In vitro studies on epidermal-growth-factor-receptor-overexpressing cell lines showed that the intracellular drug delivery by the liposomes was 3-fold higher. In a CRC xenograft model, drug delivery revealed a strongly enhanced treatment outcome with a superior drug accumulation in the tumour tissue of 2916.0 ± 507.84 ng/g compared to cetuximab liposomes (1546.02 ± 362.41 ng/g) or non-targeted liposomes (891.06 ± 155.1 ng/g) [113]. The liposomal formulation was effective on all the epidermal-growth-factor-receptor-overexpressing cell lines and also reversed oxaliplatin resistance and sensitivity. The advantage of liposome formulations is their selective uptake and enhanced intracellular drug concentration [113].

Tummala et al. designed hybrid liposomal nanoparticles loaded with oxaliplatin and tumour-necrosis-factor-related apoptosis-inducing ligand for the treatment of CRC [114]. The overexpression of the protein of tumour-necrosis-factor-related apoptosis-inducing ligand in tumour cells makes it a specific site for drug delivery. The formulation was composed of lipid and polymer loaded with oxaliplatin and antitumour-necrosis-factor-related apoptosis-inducing ligand antibody for targeted drug delivery. The polymeric core was prepared from chitosan for encapsulating oxaliplatin and the outer lipid layer was prepared from soya lecithin and cholesterol, and DSPE-PEG-2000 and used for the covalent incorporation of Anti-TRAIL. The polymer layer was effective for the sustained drug release at the target site while the lipid layer enhanced the stability of the formulation and targeted drug delivery. The formulation decreased the tumour mass and volume in vivo in xenograft tumour models. The drug release profile from the formulation was sustained. The formulation is a potential treatment for colorectal cancer [114]. Liu et al. packaged miR-128-3p onto secreted exosomes using miR-128-3p-transfected cells [115]. Injecting these miR-128-3p-loaded exosomes into oxaliplatin-resistant CRC cells efficiently delivered miR-128-3p, according to the scientists. In oxaliplatin-resistant CRC cells, exosome-assisted miRNA delivery reduced tumour expansion and enhanced oxaliplatin responsiveness, suggesting a viable therapeutic option for oxaliplatin-resistant CRC patients [115]. Yang et al. looked into the antitumor activity of PEG liposomal l-OHP in a xenograft tumour-bearing nude mouse model [116]. In contrast to free l-OHP, they established the intravenous treatment of PEG-liposomal l-OHP increased l-OHP accumulation in tumour tissues through the leaky tumour vasculature via the EPR effect, resulting in a significant reduction in tumour burden and increased mouse longevity. They proposed that PEG-liposomal l-OHP could be a good substitute for free l-OHP in the treatment of CRC [116]. Liu et al. looked into the possibility of using extremely sensitive sPLA2-responsive liposomes as drug carriers for encapsulating the platinum-based medication oxaliplatin (L-OHP) for colon cancer treatment. L-OHP has been demonstrated to be particularly helpful in the treatment of colorectal malignancies while also being free of nephrotoxicity [117]. Yang et al. reported that several agents under preclinical studies displayed promising in vitro results with potential applications for the treatment of CRC, together with oxaliplatin-loaded long-circulating liposomes, which include PEG-liposomal L-oHP [118].

Liposomes loaded with oxaliplatin possess promising therapeutic outcomes against CRC cell lines because they can induce a significant apoptotic reaction against a human colorectal cancer cell line, exhibit good drug delivery in vivo, and reduce tumour growth. Liposomes loaded with oxaliplatin improved oxaliplatin responsiveness with increased accumulation in the tumour tissues. In vivo studies also revealed a longer survival rate with reduced nephrotoxicity. Liposomes loaded with oxaliplatin have a promising potential application for the treatment of CRC.

#### 4.3.5. Polymeric Nanoparticles (PNPs) Loaded with Oxaliplatin

**Figure 5:**
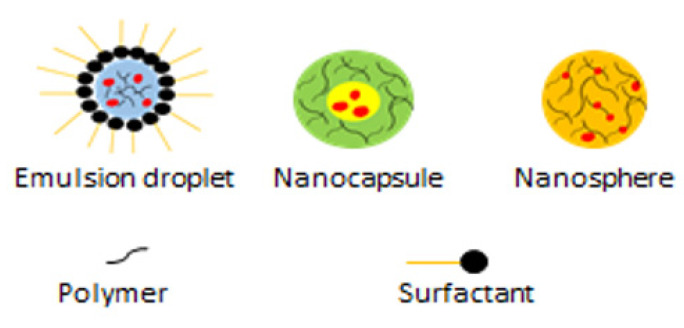
Polymeric Nanoparticles.

PNPs are particles or particulate materials with a one-dimensional size of approximately 10–1000 nm (Figure 5) [119]. PNPs have been employed for a variety of applications in numerous biomedical fields due to their incredibly high volume–surface area ratio, tunable pore size, and small size [120].

Guo et al. loaded a combination of oxaliplatin and folinic acid into an aminoethyl anisamide PEGylated lipid nanoparticle inside a microemulsion via a nanoprecipitation technique. The formulation exhibited interesting features including extended blood circulation and enhanced accumulation in the tumour in an orthotopic CRC mouse model. A significant chemo-immunotherapeutic response resulting from the combination of the formulation and 5-fluorouracil was not associated with toxicity [121]. Wang et al. loaded oxaliplatin into D-α-Tocopherol polyethylene glycol 1000 succinate-based lipid nanoparticles with an increased anticancer effect in HT-29 colon cancer cells. The IC_50_ value of the formulation was 1.12 μg/mL compared to the free drug (4.25 μg/mL). The formulation induced significant apoptosis of the cancer cells with 52% early apoptosis phase and 13% late apoptosis phase [122]. Luiza et al. evaluated the anticancer efficacy of retinoic acid and oxaliplatin loaded into cholesterol-coated Poly (*D*, *L*-lactide-co-glycolic acid) NPs for effective encapsulation and administration [123]. The drug-loaded nanoparticles reduced the proliferation of tumoral cell lines (CT-26 and SW-480) and the viability in vitro as contrasted to controls. The findings reveal that loading oxaliplatin in NP formulation with retinoic acid and cholesterol allows for optimal anticancer activity [123].

Narmani et al. used nanocarrier technology with improved targeting effectiveness against folic acid receptor-expressing colorectal cancer cells in vitro and investigated the anticancer activity of oxaliplatin [124]. Poly(amidoamine) (PAMAM) dendrimers G4 imprinted together with folic acid and polyethylene glycol increased the half-life and stability, together with nonantigenic properties and the nonimmunogenic effect. In vitro studies on the SW480 cell line revealed the PEG-PAMAM nano-complex loaded with oxaliplatin displayed a high cellular uptake of 84.67% and inhibited tumour growth. The cell viability of the SW480 cell line after treatment with the formulation was 18.39% with 81.8% late apoptotic phase in vitro [124,125].

Gowda et al. investigated oxaliplatin microspheres loaded into capsules and coated with pH-sensitive polymer for the treatment of colon cancer via oral administration. The in vitro drug release was 90.36 % in 24 h with a controlled drug release profile appropriate for enhanced therapeutic efficacy, reduced toxicity, and improved survival rate. The formulation is an effective alternative to the intravenous route [126]. Maspes et al. reviewed different polymers and their therapeutic efficacy when used for the development of nanoparticle formulations loaded with anticancer drugs for the treatment of CRC [127].

Duan et al. reported the potential of NPs to stimulate the tumour microenvironments thereby inducing antitumour immunity. A nanoscale coordination polymer core–shell particle was loaded with a combination of oxaliplatin and dihydroartemisinin. The formulation improved the uptake of the drug into the tumour and induced immunostimulatory properties. It also promoted cancer cell phagocytosis and, in vivo, the animal models were free from the tumour for 3 months and immunized against live tumour cells showing the efficacy of combination therapy in activating the innate and adaptive immune systems, resulting in long-lasting antitumor immunity [128,129]. Jain et al. prepared hyaluronic acid–chitosan NPs loaded with oxaliplatin which was encapsulated into Eudragit S100-coated pellets for drug delivery to colon tumours. In vivo study was performed and the formulation was administered orally at the dose of 10 mg per kg body weight to tumour-bearing Balb/c mice. Amounts of 1.99 ± 0.82 and 9.36 ± 1.10 μg of the drug were loaded into the colon tissue and tumour, respectively, over 12 h, revealing a high drug uptake into the colon tumours. The coupling of hyaluronic acid to the NP surfaces promoted targeted drug delivery to the colon tumour tissues. Drug targeting and delivery preserves cytotoxicity and reduces toxicity to normal healthy tissues, resulting in improved therapeutic efficacy and safety [130,131]. Hassanzadeganroudsari et al. synthesized oxaliplatin-loaded NPs via reverse-phase evaporation coated with the hydrophilic polymer, polyethylene glycol. The zeta potential and size of nanoparticles were −15.81.4 mV and 171.730 nm. Over 95% of the drug was released in 10 h. Loading the drug in the NPs enhanced the drug stability and plasma half-life and reduced the side effects [132]. Urbanska et al. incorporated oxaliplatin in lipid-like PNPs that were then enclosed in mucoadhesive micro-sized alginate-based particles [133]. In a CRC orthotopic mouse model, the in vivo data revealed enhanced survival and lower tumour development after 17 weeks of oral administration of the formulation compared to the control group [133]. Yang et al. reviewed lipid-based core–shell polymer nanoparticles for enhanced drug uptake into the tumour and the inhibition of tumour recurrence [134].

The anticancer drugs loaded into PNPs accumulated in the tumour with extended blood circulation. They induced significant apoptosis of the cancer cells. In vitro evaluation of PNPs loaded with oxaliplatin showed reduced proliferation and viability of CT-26 and SW-480 cell lines. PNPs loaded with oxaliplatin control the rate of drug release into the colon and are effective against CRC and its metastases. They also lower the risk of peripheral neuropathy caused by oxaliplatin as well as activate adaptive immune systems and innately lead to long-lasting antitumor immunity. They are capable of reducing the side effects and maintaining and enhancing the survival rate in vivo and lowering tumour development in mouse models. PNPs loaded with oxaliplatin increased drug concentration in the colonic milieu and colonic tumours, preventing tumour recurrence. The PNPs loaded with oxaliplatin demonstrated great potential in the treatment of CRC.

#### 4.3.6. Carbon Nanotubes (CNTs) Loaded with Oxaliplatin

CNTs are carbon-based biomaterials [135]. They are graphite tubes with a tubular form. Single-walled carbon nanotubes (SWCNTs) have unique physicochemical features that boost their performance as nanocarriers and allow them to be used in a wide range of applications, such as high surface area, rich electronic polyaromatic structure, excellent chemical stability, ability to adsorb therapeutic molecules, etc. The drugs can either be loaded into the CNT structure or attached to the surface for delivery via the endocytosis pathway or the diffusion pathway [136,137]. They occur as SWCNTs, double-walled carbon nanotubes (DWCNTs), and multiwalled carbon nanotubes (MWCNTs) [138].

#### 4.3.7. Multiwalled Carbon Nanotube Loaded with Oxaliplatin

MWCNTs’ diameter ranges from 3 to 30 nm and they can expand to be several centimetres long; therefore, their aspect ratio can range from ten to ten million [139]. MWCNTs have been employed for the design of drug delivery systems for the targeted delivery of platinum drugs. Lee et al. encapsulated oxaliplatin into PEGylated MWNTs coated with superparamagnetic iron oxide for magnetic resonance imaging to evaluate the prolonged drug release suitable for reducing platinum drug biotoxicity [140]. Only 36.25% of the loaded oxaliplatin was released within 12 h, whereas 55.48% was released beyond 144 h, demonstrating the potential of the formulation to provide a prolonged drug release. In vivo studies showed that the formulation antitumor activity was comparable to the free drug treatment with no significant side effects [140].

Wu et al. incorporated oxaliplatin into the inner cavity of PEGylated multiwalled carbon nanotubes via nanoextraction. The release of oxaliplatin was sustained release with 34% of oxaliplatin released in 6 h. The formulation’s cytotoxic effect was reduced at 12 and 24 h on HT-29 cell lines but increased significantly at 48 and 96 h, attributing to the sustained drug release profile of the formulation [141]. The MWCNT-based formulations loaded with oxaliplatin displayed sustained release. In vitro results demonstrated an improved cytotoxic effect in vivo and in vitro revealed good antitumour activity. Their uptake into the cancer tumour is a crucial feature for the management of colorectal cancer.

#### 4.3.8. Metal Nanoparticles Loaded with Oxaliplatin

Metal NPs have great potential applications in both medical and nonmedical fields [142]. Metallic NPs are currently used widely in biomedical and engineering fields [143]. They are also designed for drug delivery due to their nanosized range and charged surface. Wheate et al. attached oxaliplatin to gold nanoparticles by chelating platinum (II) species to gold nanoparticles that were functionalized with a thiolated poly(ethylene glycol) (PEG) monolayer closed with a carboxylate group [144]. The formulation effects on colon cancer cell lines (HCT116, HCT15, HT29, and RKO) in vitro were 5.6-fold more cytotoxic or similar to the free oxaliplatin. The uptake of the NP was via endocytosis [144,145].

Gholami et al. incorporated copper sulfide into a UiO-66-NH_2_ delivery system loaded with oxaliplatin for the treatment of colorectal cancer. The invitro cytotoxicity assay revealed the efficacy of the drug delivery system against colorectal cancer cell lines. The CuS acted synergistically with the loaded drug [146]. Jabalera et al. investigated the potential of the oxaliplatin–biomimetic magnetic NPs for targeted chemotherapy against CRC [147]. The nanoformulation was stable under physiological conditions with 20% drug release in one hour, good cytocompatibility, and increased cytotoxic effect in the colon cancer cells.

## 5. Commercially Available Nanomaterials for Colorectal Cancer Therapy

Along with its cytotoxicity and adverse effects on normal tissues, traditional chemotherapeutic drugs used for the treatment of CRC have inadequate efficacy [148,149]. As a result, new, effective, and safe CRC treatments are needed. When compared with untreated drug forms, NP-mediated formulations enhanced the therapeutic efficacy and reduced adverse effects [150]. The utilization of NPs as delivery vehicles has several benefits: (1) it improves drug stability and solubility in harsh GIT conditions; (2) it extends the half-life of drug payloads in blood circulation; (3) it enhances permeability and retention (EPR) effect in tumour lesions; thus, the passive targeting capacity is expected to augment intratumoral drug accumulation, which is required for enhanced efficacy [151]; (4) it overcomes the mechanism of drug resistance in malignant cells, therefore diminishing the concentration of the therapeutics needed for treatment [151]; (5) it improves stability and avoids in vivo decomposition [152]; (6) it can encapsulate multiple drugs and target specific sites to fabricate good therapeutic effects [153,154]; and (7) it reduces toxicity. Several NPs that induce apoptosis has been tested in preclinical studies on CRC models, although the majority of them are not clinically licensed (Table 1).

## 6. Conclusions

The use of different nanoparticles such as micelles, gold NPs, liposomes, polymeric NPs, phytosomes, dendrimers, magnetic NPs, etc., to load platinum drugs resulted in promising anticancer activity for the treatment of CRC. There are several reports on nanoparticles’ capability to enhance drug–water solubility and enable disease-specific transportation of the loaded drugs, reduce toxicity, and promote targeted drug delivery. Platinum drugs have also been used in combination with other cytotoxic drugs including doxorubicin, paclitaxel, and gemcitabine. These nanoparticles are promising platforms used for combination therapy with controlled drug release profiles of individual drugs. Although the incorporation of platinum drugs into these NPs offers a promising effect, some of the systems loaded with platinum drugs were found to be less potent compared to the free drugs against CRC cells, suggesting that the design of the formulation may have reduced the anticancer activity of the loaded drug. The nanoparticles should also be designed in a manner to address the tumour biology by enhancing drug uptake because the characteristics and the microenvironment of the tumour differ for each patient. There is a pressing need to improve the design of nanoparticle carriers to promote the release of the required amount of the loaded drug over a sufficient period that will enhance the anticancer effects. A thorough investigation into the toxicological impact of the nanoparticles over a prolonged period should be undertaken. More in vivo preclinical studies are needed to fully understand the mode of action of the formulations against CRC. The design of nanoparticles for drug delivery offers great potential in increasing the quality and life expectancy of CRC patients, but more research is needed.

## Figures and Tables

**Table 1:** Some nanoformulations have been prepared for the treatment of colorectal cancer.

Nanoparticle Formulations	Drugs Used	Type of Nanoparticles	References
FOLFOX	5-FU, Oxaliplatin	Lipid-based NPs	[121]
5-FU/PEG-PBLG	5-FU	Polymeric NPs	[155]
HACTNP	5-FU	Polymeric NPs	[156]
Xyl-SA/5-FUSA	5-FU	Conjugate NPs	[157]
CPX-1	Irinotecan HCl	Liposomes	[158]
SLNPs containing 5-FU	5-FU	Solid NPs	[159,160]
5-FU/GSH-GNPs	5-FU	Conjugate NPs	[161]
Chitosan-HA-Oxa NPs	Oxaliplatin	Polymeric NPs	[153]
Oxaliplatin encapsulated in chitosan-coated alginate microspheres	Oxaliplatin	Polymeric NPs	[133]
PEG-liposomal L-oHP	Oxaliplatin	Polymeric NPs	[116]
Liposome-embedding silicon microparticle	Oxaliplatin	Liposomes	[162]
Nanoscale coordination polymer (NCP) core–shell particles	Oxaliplatin, DHA	Liposomes	[128]
pH-responsive PEG-shedding and targeting Peptide-modified nanoparticles	Irinotecan, miR-200	Polymeric NPs	[163]
Lipid bilayer-coated MSNP carrier	Irinotecan	Liposomes and Polymeric NPs	[164]
Liposomal irinotecan (Lipo-IRI)	Irinotecan	Liposomes	[165]
SN38 (LA-SN38)-loaded NPs	SN38	Lipid-based NPs	[166]
CD133Ab-NPs-SN-38	SN38	Polymeric NPs	[167]
nSN38	nCUR SN38, curcumin	Conjugated NPs	[168]
PLGA-PTX	Paclitaxel	Polymeric NPs	[169]
Paclitaxel-loaded magnetic nanocarriers	Paclitaxel	Polymeric NPs	[170]
Celecoxib-containing Hap-Cht NPs	Celecoxib	Conjugated NPs	[171]
Chitosan NPs	Gemcitabine, curcumin	Polymeric NPs	[172,173]
WGA-conjugated PLGA NPs loaded with Pac	Paclitaxel	Conjugated NPs	[174]
Aspirin-loaded nanoexosomes	Aspirin	Conjugated NPs	[175]
A33Ab-US-Exo/Dox	Doxorubicin	Conjugated NPs	[176]
EGFR-targeted evodiamine NPs	Evodiamine	Polymeric NPs	[177]
miR-139-5p-EpCAM Apt-HSPC/DOTAP/Chol/DSPE-PEG2000-COOH nanoparticles	MANPs miR-139-5p	Liposomes	[178]
Chol-butyrate SLNP formulation	Butyric acid	Lipid-based NPs	[179]
PEG-PLGA-endostar	Endostatin	Polymeric NPs	[180]
Hafnium oxide nanoparticles (NBTXR3)	-	NBTX 3	[181]
Silver nanoparticles (AgNPs)	-	Metallic NPs	[182]
PEG-AuIONs	-	Polymeric NPs	[183]

## Abbreviations

DNA	Deoxyribonucleic acid
CTR1	Copper transport protein
MSN	Medium spiny neurons
PLGA	Poly D,L-lactic-co-glycolic acid
LNPs	Lipid nanoparticles
PAMAM	Polyamidoamines
PEG	Polyethylene glycol
PNP	Psychoneuroplasticity
SWCNTs	Single-walled carbon nanotubes
FU	Fluorouracil
HACTNPs	Hyaluronic acid Coupled Chitosan Nanoparticles
CPX-	Ciprofloxacin
SLNPs	Solid lipid nanoparticles
TME	Tumour microenvironment
NSCLC	Non-small cancer lung cell
SWCNTs	Single-walled carbon nanotubes
